# Parity–time-symmetric circular Bragg lasers: a proposal and analysis

**DOI:** 10.1038/srep37688

**Published:** 2016-11-28

**Authors:** Jiahua Gu, Xiang Xi, Jingwen Ma, Zejie Yu, Xiankai Sun

**Affiliations:** 1 Department of Electronic Engineering, The Chinese University of Hong Kong, Shatin, New Territories, Hong Kong; 2 Shun Hing Institute of Advanced Engineering, The Chinese University of Hong Kong, Shatin, New Territories, Hong Kong

## Abstract

We propose a new type of semiconductor lasers by implementing the concept of parity–time symmetry in a two-dimensional circular Bragg grating structure, where both the real and imaginary parts of the refractive index are modulated along the radial direction. The laser modal properties are analyzed with a transfer-matrix method and are verified with numerical simulation of a practical design. Compared with conventional distributed-feedback lasers with modulation of only the real part of refractive index, the parity–time-symmetric circular Bragg lasers feature reduced threshold and enhanced modal discrimination, which in combination with the intrinsic circularly symmetric, large emission aperture are clear advantages in applications that require mode-hop-free, high-power, single-mode laser operation.

Semiconductor lasers are an important building block in fiber-optic communication, where lasers of pure output spectrum, compact size, high reliability, and low cost are usually desired^1^. Many efforts have been made to obtain high-performance lasers. For example, distributed-feedback and distributed-Bragg-reflector structures have been developed for creating large discrimination in threshold gain among different oscillation modes of a laser cavity, which facilitates the realization of single-mode lasers^2^,^3^. In the meantime, quantum well and quantum dot structures have been employed for improving power efficiency and thermal stability^4^. Circular Bragg lasers constructed by cylindrical distributed Bragg reflectors were studied decades ago, where high-*Q*-factor, large-area, single-mode laser emission can be obtained in a broad operation range^5^,^6^,^7^,^8^. With the intrinsic circular aperture and low-divergence emission angle, such lasers have advantages in coupling their emitted light directly into an optical fiber or to on-chip photonic components, thus lending themselves to a wide range of applications in integrated photonics, optoelectronics, and fiber-optic communication.

The concept of parity–time (*PT*) symmetry was first developed by Bender *et al*. in quantum mechanics^9^. A Hamiltonian is called *PT* symmetric if it commutes with the *PT* operator, which requires that the real (imaginary) part of the complex potential be an even (odd) function of the coordinates. It was found later that this concept also applies to optical systems due to the resemblance between the Schrödinger equation and the wave equations^10^. *PT* symmetry in optics can be realized similarly by introducing modulation to both the real and imaginary parts of the refractive index, where the modulation pattern follows an even and odd function respectively^11^. This trick has been implemented in several photonic structures to achieve otherwise unattainable functionalities, such as lasers and laser amplifiers^12^,^13^,^14^,^15^,^16^,^17^,^18^, coupled nanobeam cavities^19^, unidirectional reflectionless^20^ and nonreciprocal transmission^21^ optical components.

In this paper, we for the first time introduce the *PT* symmetry into the design of circular Bragg lasers. By using a transfer-matrix method, we first analyze the reflection and transmission properties of the *PT*-symmetric circular Bragg reflectors (CBRs), from which the *PT*-symmetric circular Bragg lasers are constructed. A comparison between the modal properties of the *PT*-symmetric circular Bragg lasers and their conventional counterparts concludes that the former possess a significantly lower threshold and larger modal discrimination for the targeted lasing mode, both of which contribute crucially to the development of mode-hop-free, single-mode lasers for high-power applications. Numerical results from finite-difference time-domain simulation of a practical design show good agreement with those from the transfer-matrix method.

## Results

### Structural description of the proposed *PT*-symmetric circular Bragg lasers

**Figure 1:**
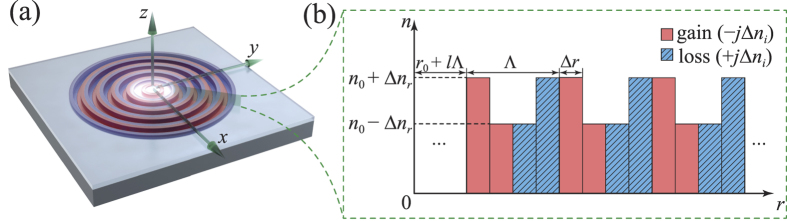
(**a**) Schematic of the proposed *PT*-symmetric circular Bragg laser, where light is confined tightly by the *PT*-symmetric circular Bragg reflector to the center region. (**b**) Radial profile of the *PT*-symmetric circular Bragg reflector, showing periodic modulation of both the real and imaginary parts of the refractive index.

Figure 1(a) illustrates the two-dimensional *PT*-symmetric circular Bragg laser on a chip. Such lasers can be fabricated from a III–V epiwafer which is able to provide optical gain under optical or electrical pumping. The *PT* symmetry is obtained in the CBR by introducing modulation to both the real and imaginary parts of the refractive index along the radial direction (*r*) as shown in Fig. 1(b), which can be realized respectively by selective etching and metal deposition on the III–V epiwafer^12^. More specifically, the complex refractive index of the CBR is expressed by


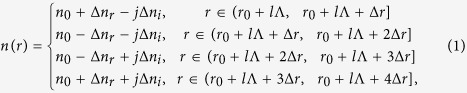


where *r*_0_ is the starting radius of the CBR and *n*_0_ is the average effective refractive index. ∆*n*_*r*_ and ∆*n*_*i*_ are the modulation depths of the real and imaginary part of the refractive index, respectively. *l* is an integer starting from 0. Δ*r* is the thickness of each modulated layer. The modulation period of the refractive index is Λ = 4·Δ*r*. With *N* periods of modulation, the CBR spans a length of *N*·Λ in the radial direction. It should be noted that the layer thickness Δ*r* generally should not be set as a constant for two reasons: First, the phase of the eigenmodes of traveling waves in the cylindrical coordinates, i.e., the Hankel functions, does not follow a linear dependence with *r*. Therefore, Δ*r* should follow the local period of the Hankel functions for obtaining perfect phase matching, thus rendering chirped modulation along the radial direction^5^,^8^. Second, the wavelength in each modulated layer is different and thus Δ*r* should be inversely proportional to the real part of the refractive index of the respective layer. In this study, the above two effects are negligible for large radius under the weak-modulation condition (∆*n*_*r*_ ≪ *n*_0_). The *PT* symmetry is satisfied under the condition that the real and imaginary parts of the refractive index are respectively an even and odd function of position along the radial direction (*r*).

### Reflection and transmission properties of the *PT*-symmetric circular Bragg reflectors

We develop a transfer-matrix method similar to that in ref. 22 for analyzing the *PT*-symmetric CBR. It is convenient to consider the optical field components satisfying the Helmholtz equation in cylindrical coordinates, which can be expressed by the *z* component of the electric and magnetic fields


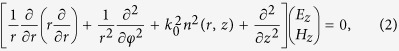


where *r*, *φ*, and *z* are the radial, azimuthal, and axial coordinates respectively, and *k*_0_ is the wavevector in vacuum. By using the effective medium approach, we can simplify the problem from three dimensional into two dimensional so that the refractive index *n*(*r*, *z*) reduces to *n*(*r*) as defined in Eq. (1) and ∂^2^/∂*z*^2^ can be dropped from Eq. (2). Assuming that the *r* and *φ* dependence of the field can be separated, we obtain *E*_*z*_(*r*, *φ*) = *E*_*z*_(*r*) exp(*jmφ*), where *m* is an integer representing the azimuthal modal number. Introducing *E*_*z*_(*r*, *φ*) into Eq. (2) leads to


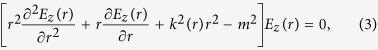


where *k*(*r*) = *k*_0_ *n*(*r*) is constant within each modulated layer. The solution of Eq. (3) can be expressed as a linear combination of the Hankel functions of the first and second kinds^23^


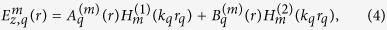


where *k*_*q*_ and *r*_*q*_ denote respectively the wavevector and radius of the *q*th layer. 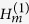 and 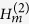 represent the outward- and inward-going cylindrical modes, with their amplitudes denoted by 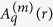 and 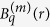 respectively. We can rewrite Eq. (4) and its derivative in a matrix form


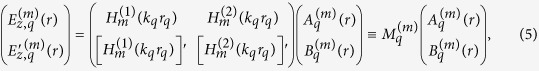


where 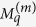 is defined as the coefficient matrix. Based on the continuity conditions of the electric and magnetic fields at each interface, the relation between Layer *q* and *q* + 1 can be expressed as


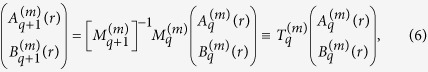


where 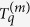 is the transfer matrix from Layer *q* to *q* + 1. As a result, by multiplying the transfer matrices of each layer we establish a relation between the amplitudes in Layer 1 and *N* + 1:


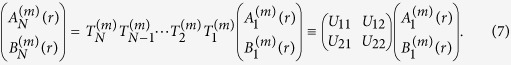


Let us consider an outward-going cylindrical wave impinging on the 1st layer of the CBR with an amplitude 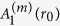. By setting 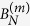
(*r*_0_ + *L*) = 0 in Eq. (7), we obtain the reflection coefficient *R* = |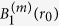/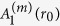
|^2^ = |−*U*_21_/*U*_22_|^2^ and the transmission coefficient *T* = |
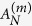
(*r*_0_ + *L*)/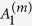
(*r*_0_)|^2^ = |*U*_11_ − *U*_12_ × *U*_21_/*U*_22_|^2^.

**Figure 2:**
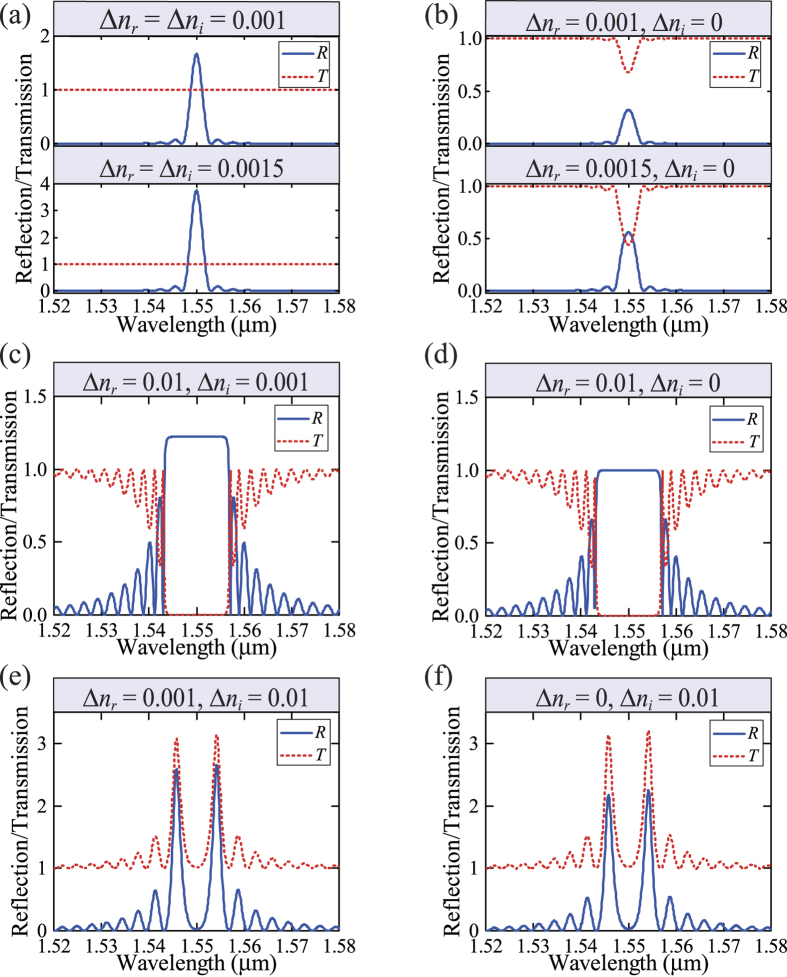
Reflection (*R*) and transmission (*T*) spectra of the *PT*-symmetric circular Bragg reflectors (CBRs) calculated by a transfer-matrix method for different modulation depths of the real and imaginary parts of the refractive index. An outward-going cylindrical wave impinges on the innermost layer at *r* = *r*_0_. (**a**) With ∆*n*_*i*_ = ∆*n*_*r*_ the CBRs operate at the spontaneous *PT*-symmetry breaking point. (**b**,**c**,**d**) With ∆*n*_*i*_ < ∆*n*_*r*_ the CBRs operate in the *PT*-symmetric phase. (**e**,**f**) With ∆*n*_*i*_ > ∆*n*_*r*_ the CBRs operate in the broken-*PT*-symmetric phase.

We aim at designing a circular Bragg laser which emits circularly symmetric beam (*m* = 0) at a vacuum wavelength *λ*_0_ of 1550 nm. Without loss of generality, we may assume the average effective refractive index *n*_0_ to be 1.55 and the resulting effective wavelength inside the CBR *λ*_eff_ = *λ*_0_/*n*_0_ = 1.00 μm. The thickness of each modulated layer Δ*r* is set to be 125 nm, and thus the modulation period Λ is 4Δ*r* = 500 nm. We choose the number of the modulation periods *N* to be 500 and the corresponding radial length *L* to be *N*·Λ = 250 μm. We investigate the reflection (*R*) and transmission (*T*) response of the *PT*-symmetric CBR to an outward-going wave impinging on the innermost layer. Figure 2(a) shows the calculation results when ∆*n*_*r*_ = ∆*n*_*i*_ = 1.0 × 10^−3^ or 1.5 × 10^−3^. It is clear that *R* can be larger than 1 at the designed wavelength and stronger modulation of the refractive index leads to enhanced *R*. These behaviors do not contradict with the conservation of energy because the *PT*-symmetric CBR structure forces more electric field to be distributed in the gain regions. Therefore, we can control the reflection strength of the CBR by designing an appropriate modulation depth. Meanwhile, the transmission *T* remains wavelength independent and always equal to 1. As a comparison, Fig. 2(b) plots the results for a traditional CBR with ∆*n*_*i*_ = 0 and ∆*n*_*r*_ = 1.0 × 10^−3^ or 1.5 × 10^−3^. Under the same modulation depth ∆*n*_*r*_, the reflection at the targeted wavelength is much weaker than that in Fig. 2(a) and is always smaller than 1. Moreover, *R* and *T* add up to 1 in accordance with the conservation of energy in the traditional sense.

It is interesting to study the behavior of *R* and *T* when ∆*n*_*r*_ and ∆*n*_*i*_ are unequal. Figure 2(c) shows the results for ∆*n*_*r*_ = 1.0 × 10^−2^ and ∆*n*_i_ = 1.0 × 10^−3^, where the modulation to the real part of the refractive index dominates. The reflection and transmission spectra are similar to those of the traditional counterpart (∆*n*_*i*_ = 0) as shown in Fig. 2(d), where the reflection for the side modes is enhanced due to the strong ∆*n*_*r*_. The only difference is that the *PT*-symmetric CBR provides overall stronger reflection, which can exceed 1 at the peak, than the traditional CBR owing to the additional modulation ∆*n*_*i*_. Figure 2(e) shows the results for ∆*n*_*r*_ = 1.0 × 10^−3^ and ∆*n*_*i*_ = 1.0 × 10^−2^, where the modulation to the imaginary part of the refractive index dominates. In this case, the reflection and transmission spectra take similar patterns where *R* is greatly suppressed at the targeted wavelength and enhanced for the side modes. These results also resemble those of a structure with pure gain modulation (∆*n*_*r*_ = 0) as shown in Fig. 2(f), although the *PT*-symmetric CBR provides overall stronger reflection owing to the additional modulation ∆*n*_*r*_. The results in Fig. 2(c–f) have clearly shown that when ∆*n*_*r*_ and ∆*n*_*i*_ are unequal, the larger of the two determines the reflection and transmission characteristics. The imbalance between ∆*n*_*r*_ and ∆*n*_*i*_ results in reflection reduction at the targeted wavelength and enhancement for the side modes, leading to worse discrimination between the designed and unwanted modes. Therefore, it is crucial to balance the ∆*n*_*r*_ and ∆*n*_*i*_ in a *PT*-symmetric CBR for designing robust single-mode lasers.

It is important to note that, under different modulation schemes in Fig. 2, the devices operate in different phases (*PT*-symmetric phase or broken-*PT*-symmetric phase). In a recent work^24^, Ge *et al*. proposed a generalized conservation relation between the transmittance and reflectance |*T* − 1| = (*R*_*L*_·*R*_*R*_)^1/2^, which can be adopted to determine the presence of *PT* symmetry and *PT*-symmetric breaking transitions: the system is in the *PT*-symmetric phase when *T* < 1, in the broken-*PT*-symmetric phase when *T *> 1, and at the spontaneous *PT*-symmetric breaking point (i.e., the exceptional point) when *T* = 1. Therefore, in Fig. 2(a) the CBRs operate at the spontaneous *PT*-symmetric breaking point with *T* = 1, because the modulation ∆*n*_*i*_ is balanced with ∆*n*_*r*_. In Fig. 2(b,c,d) the CBRs operate in the *PT*-symmetric phase with *T* < 1, because the modulation ∆*n*_*i*_ is trivial compared with ∆*n*_*r*_. In Fig. 2(e,f) the CBRs operate in the broken-*PT*-symmetric phase with *T* > 1, because the modulation ∆*n*_*i*_ is larger than ∆*n*_*r*_.

### Modal analysis of the *PT*-symmetric circular Bragg lasers

**Figure 3:**
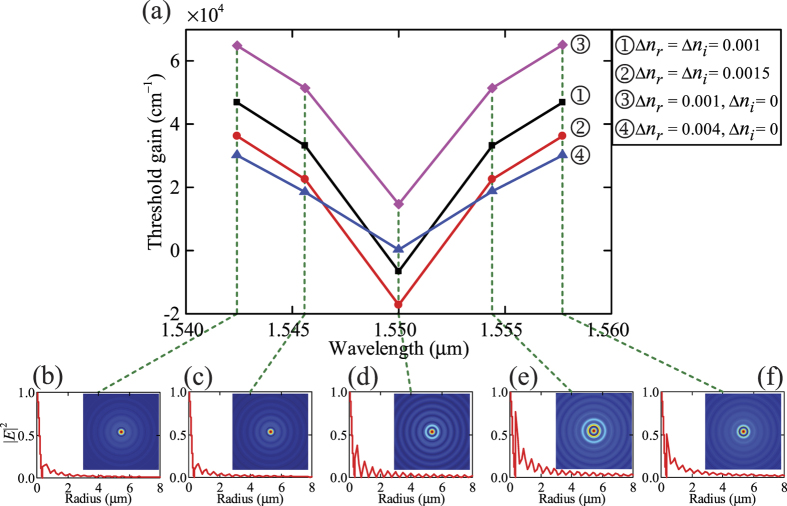
(**a**) Threshold gain of lasing modes with different modulation depths of the real and imaginary parts of the refractive index. The *PT*-symmetric circular Bragg lasers with refractive index modulation profiles ① and ② operate at the spontaneous *PT*-symmetric breaking point. The traditional circular Bragg lasers with refractive index modulation profiles ③ and ④ operate in the *PT*-symmetric phase. (**b**–**f**) |*E*|^2^ field distributions of the first five lasing modes in (**a**).

Now we analyze a laser structure constructed from the *PT*-symmetric CBRs. The laser structure consists of a central disk-shaped gain or loss region surrounded by a *PT*-symmetric CBR. It should be noted that the proposal of *PT*-symmetric laser structures does not have limitation on the average effective refractive index *n*_0_. One can always design the structural parameters (e.g., the CBR’s starting radius *r*_0_ or the thickness of each modulated layer Δ*r*) based on a specific material system to satisfy the laser oscillation condition and obtain perfect phase matching at the targeted wavelength. The laser oscillation condition is *r*_CBR_ · *r*_ctr_ · *δ*_disk_ = 1, where *r*_CBR_ = 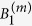
(*r*_0_)/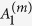
(*r*_0_) is the complex reflection coefficient of the CBR which depends on the modulation depths of the real and imaginary parts of the refractive index. *r*_ctr_ is the reflection coefficient at the center of the disk which must be exactly 1 in order to keep the finiteness of the total field. *δ*_disk_ is a complex propagation factor expressed as exp[2 *g*(*λ*)·*r*_0_ + 2*j*·*ϕ*(*λ*)], which contains the amplitude and phase information of light propagating radially in the central disk. *g*(*λ*) and *ϕ*(*λ*) represent the wavelength-dependent gain/loss coefficient and the phase change, respectively. To satisfy the laser oscillation condition, the radius of the central disk region must be chosen such that light at the targeted wavelength *λ*_0_ experiences a phase change *ϕ* of multiple integers of 2π. Therefore, we choose *r*_0_ to be 380 nm which corresponds to the first zero of the Bessel function of the first kind^25^. The light propagating in the central disk region experiences either a gain or a loss depending on the sign of *g*(*λ*) in *δ*_disk_. From the laser oscillation condition we can obtain *g*(*λ*) for each mode, which is the threshold gain required for lasing. The threshold gain of the first five lasing modes under different refractive index modulation is plotted in Fig. 3(a), while their one- and two-dimensional modal field distributions are presented in Fig. 3(b–f). For lasers constructed from the *PT*-symmetric CBRs as shown in ① and ② where ∆*n*_*r*_ = ∆*n*_*i*_ = 1.0 × 10^−3^ or 1.5 × 10^−3^, we find a negative threshold gain for the targeted wavelength, indicating that no additional gain is necessary for the targeted mode to lase and the lasing can occur even when the central disk region is lossy. The difference between the threshold gain of the targeted mode and its adjacent modes is as high as 3.99 × 10^4^ cm^−1^, yielding excellent modal discrimination for single-mode laser operation. Moreover, increase in the modulation depths leads to a uniform reduction of threshold gain for all the modes, and thus the large modal discrimination is maintained. In contrast, lasers constructed from conventional CBRs with ∆*n*_*i*_ = 0 as shown in ③ and ④ always require a positive threshold gain at the targeted wavelength, no matter how strong the modulation depth ∆*n*_*r*_ is. For ∆*n*_*r*_ = 1.0 × 10^−3^ as shown in ③, the threshold gain is 1.47 × 10^4^ cm^−1^ and the modal discrimination is 3.68 × 10^4^ cm^−1^. Although the threshold gain of the targeted mode can be reduced by increasing ∆*n*_*r*_, e.g., from 1.0 × 10^−3^ to 4.0 × 10^−3^, this results in greater reduction of threshold gain of the unwanted modes, causing worse modal discrimination (e.g., 1.81 × 10^4^ cm^−1^ in ④) and thus less robust single-mode laser operation. Therefore, we conclude that *PT*-symmetric circular Bragg lasers have clear advantages over their conventional counterparts because the former possess much lower threshold gain and larger modal discrimination, both of which facilitate the realization of single-mode lasers.

**Figure 4:**
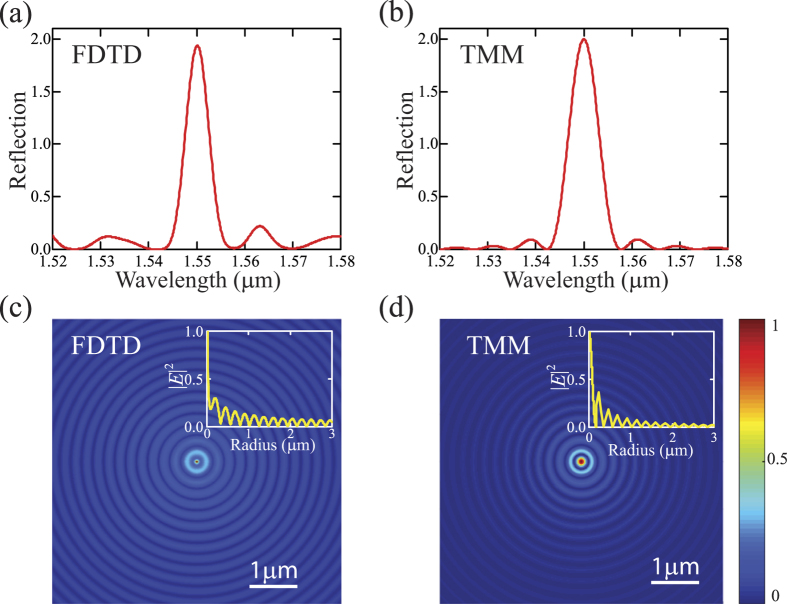
(**a**,**b**) Reflection spectra of an outward-going cylindrical wave impinging onto a *PT*-symmetric circular Bragg reflector, obtained from the finite-difference time-domain (FDTD) simulation (**a**) and the transfer-matrix method (TMM) (**b**). (**c**,**d**) One- and two-dimensional |*E*|^2^ field distributions of the lasing mode (*λ*_0_ = 1550 nm) obtained from the FDTD simulation (**c**) and the TMM (**d**).

In order to verify the modal analysis from the transfer-matrix method, we simulated a practical design of *PT*-symmetric circular Bragg laser based on the parameters of a quantum well wafer used previously^26^. We set the refractive index *n*_0_ and the modulation depths (∆*n*_*r*_, ∆*n*_*i*_) to be 3.40 and 0.006 respectively to satisfy the requirement of *PT* symmetry. The CBR’s starting radius *r*_0_ is 175 nm and the thickness of each modulated layer Δ*r* is 57 nm. The number of the modulation periods *N* is set to be 100, and thus the corresponding radial length *L* is 22.8 μm. It should be noted that the choice of the number of the modulation periods is related to the preset modulation depths. Smaller modulation depths can also be adopted at the expense of increased number of the modulation periods with correspondingly longer radial length^20^. Figure 4(a) shows the simulated reflection spectrum of the *PT*-symmetric CBR by using the finite-difference time-domain (FDTD) method in Lumerical Solutions^27^, which is in good agreement with that calculated from the transfer-matrix method (TMM) in Fig. 4(b). This indicates that a practical *PT*-symmetric CBR structure can be engineered for realizing single-mode lasers. We also obtained the optical field distribution from the FDTD simulation and the TMM as shown in Fig. 4(c) and (d) respectively. It is clear that light of the targeted wavelength (*λ*_0_ = 1550 nm) is confined to the central disk region thus facilitating low-threshold lasing.

## Conclusion

In conclusion, we have proposed two-dimensional parity–time-symmetric circular Bragg lasers and analyzed their modal properties including threshold gain and field distribution. Such lasers are constructed from a type of circular Bragg reflectors whose refractive index is modulated in both the real and imaginary parts along the radial direction. By setting balanced modulation depth to the real and imaginary parts we can obtain significantly reduced threshold gain with large modal discrimination for the targeted mode, facilitating robust single-mode laser operation. To demonstrate the feasibility for real applications, we also performed finite-difference time-domain simulation of a laser structure with practical design parameters, and obtained the results in good agreement with those from the transfer-matrix method. Featuring low threshold and robust single-mode operation in addition to the intrinsic circular aperture and low-divergence emission angle, such parity–time-symmetric circular Bragg lasers will find wide applications in integrated photonics, optoelectronics, and fiber-optic communication.

## Methods

The proposed *PT*-symmetric circular Bragg lasers can be fabricated from a III–V epiwafer. The modulation of both the real (∆*n*_*r*_) and imaginary (∆*n*_*i*_) parts of the refractive index along the radial direction can be realized respectively by selective etching and metal deposition on the III–V epiwafer. The performance of the *PT*-symmetric CBRs with various modulation depths (∆*n*_*r*_, ∆*n*_*i*_) is investigated with a transfer-matrix method derived in Eqs (1)–(7). To realize a practical *PT*-symmetric circular Bragg laser, the refractive index *n*_0_ and the modulation depths are set to be 3.40 and 0.006, respectively. The CBR’s starting radius is 175 nm and the thickness of each modulated layer Δ*r* is 57 nm. The number of the modulation periods *N* is set to be 100, and the corresponding radial length *L* is 22.8 μm. We employ the FDTD method in Lumerical Solutions and the transfer-matrix method to obtain the reflection spectrum of an outward-going cylindrical wave impinging onto a *PT*-symmetric CBR as well as the |*E*|^2^ field distribution of the lasing mode (*λ*_0_ = 1550 nm). In the FDTD simulation, perfectly matched layers are set as the boundary condition for the computation in Lumerical Solutions.

## Additional Information

**How to cite this article**: Gu, J. *et al*. Parity–time-symmetric circular Bragg lasers: a proposal and analysis. *Sci. Rep.* **6**, 37688; doi: 10.1038/srep37688 (2016).

**Publisher's note:** Springer Nature remains neutral with regard to jurisdictional claims in published maps and institutional affiliations.
